# Evaluation of pancreatic parenchyma proliferation into synthetic matrix implant in diabetic and non diabetic mice

**DOI:** 10.1186/1758-5996-7-S1-A159

**Published:** 2015-11-11

**Authors:** Luciana Xavier Pereira, Simone Aparecida de Almeida, Laura Alejandra Ariza Orellano, Celso Tarso Rodrigues Viana, Silvia Passos Andrade, Paula Peixoto Campos

**Affiliations:** 1 Universidade Federal de Minas Gerais, Belo Horizonte, Brazil

## Background

Biomaterials has been the focus of diversity studies in medicine regenerative area. It's important that they can providing a microenvironment of interaction of cells and extracellular matrix. Synthetic matrix of polyether polyurethane has been used by our research group as a biomaterial applied in the study of angiogenesis, inflammation and tissue repair.

## Objectives

Assess the growth of pancreatic parenchyma into synthetic matrix implant in diabetic and no diabetic mice and the associated angiogenic/inflammatory parameters.

## Materials and methods

The diabetes induction it was performed by streptozotocin administration. The synthetic matrix was implanted next the pancreas of animals (diabetics and control) and 20 days after, they were collected and processed for histological, immunohistochemical and biochemical analysis.

## Results

The proliferation of pancreatic parenchyma was observed into implants in both groups. In control group there was more cells of pancreatic islet immunostaining to insulin into implant. The diabetic group showed lower number of blood vessels but not VEGF levels. The inflammatory parameters assessed (TNF-α and CCL-2) it was higher in diabetic group than control. The number of foreign body giant cells decreased after diabetes.

## Conclusions

Despite the diabetic state seems alters some characteristics of tissue that growth into implant, the synthetic matrix used support a favorable microenvironment to proliferation of pancreatic parenchyma in vivo.

